# The relationship between regional medical campus enrollment and rates of matching to family medicine residency

**DOI:** 10.36834/cmej.69328

**Published:** 2020-07-15

**Authors:** Dorothy Bakker, Christopher Russell, Mary Lou Schmuck, Amanda Bell, Margo Mountjoy, Rob Whyte, Lawrence Grierson

**Affiliations:** 1 Department of Family Medicine, Faculty of Health Sciences, McMaster University, Ontario, Canada; 2 Michael G DeGroote School of Medicine, Faculty of Health Sciences, McMaster University, Ontario, Canada; 3 McMaster Community and Rural Education (Mac-CARE) Program, Faculty of Health Sciences, McMaster University, McMaster Community and Rural Education, Ontario, Canada; 4 McMaster Program for Education Research, Innovation, and Theory (MERIT), Faculty of Health Sciences, McMaster University, Ontario, Canada

## Abstract

**Background:**

The Michael G. DeGroote School of Medicine expanded its medical education across three campus sites (Hamilton, Niagara Regional and Waterloo Regional) in 2007. Ensuring the efficacy and equivalency of the quality of training are important accreditation considerations in distributed medical education. In addition, given the social accountability mission implicit to distributed medical education, the proportion of learners at each campus that match to family medicine residency programs upon graduation is of particular interest.

**Methods:**

By way of between campus comparisons of Canadian Residency Matching Service (CaRMS) match rates, this study investigates the family medicine match proportion of medical students from McMaster’s three medical education campuses. These analyses are further supported by between campus comparisons of Personal Progress Index (PPI), Objective Structured Clinical Examination (OSCE), Medical Council of Canada Qualifying Examination-Part 1 (MCCQE1) performances that offer insight into the equivalency and efficacy of the educational outcomes at each campus.

**Results:**

The Niagara Regional Campus (NRC) demonstrated a significantly greater proportion of students matched to family medicine. With respect to education equivalency, the proportion of students’ PPI scores that were more than two SD below the mean was comparable across campuses. OSCE analysis yielded less than 2% differences across campuses with no differences in the last year of training. The MCCQE1 pass rates were not statistically significant between campuses and there were no differences in CaRMS match rates. With respect to education efficacy, there were no differences among the three campuses’ pass rates on the MCCQE1 and CaRMS match rates with the national rates.

**Conclusions:**

Students in all campuses received equivalent educational experiences and were efficacious when compared to national metrics, while residency matches to family medicine were greater in the NRC. The reasons for this difference may be a factor of resident and leadership role-models as well as the local hospital and community environment.

## Abstract (fr)

**Contexte:**

Le campus Michael G. DeGroote School of Medicine a élargi ses programmes pré-doctoraux sur trois campus (Hamilton, Niagara Regional, et Waterloo Regional) en 2007. Aux fins de l’agrément, il est important de s’assurer de l’efficacité et de l’équivalence dans la qualité des formations dans les programmes décentralisés. De plus, vu la mission de responsabilité sociale implicite à l’éducation médicale décentralisée, la proportion de gradués jumelés à un programme de résidence en médecine familiale sur chaque campus est d’un intérêt certain.

**Méthodes:**

En comparant les taux de jumelage du Service canadien de jumelage des résidents (CaRMS) des différents campus, cette étude analyse la proportion des jumelages en médecine familiale des étudiants des des trois campus de l’Université McMaster offrant un programme de formation en médecine. Ces analyses s’appuient aussi sur les comparaisons entre ces trois campus de l’indice de progrès personnel (PPI), de l’examen clinique objectif structuré (ECOS), de l’examen d’aptitude du Conseil médical du Canada, partie I (EACMC1) qui offrent un aperçu de l’équivalence et de l’efficacité des résultats d’apprentissage à chacun de ces campus.

**Résultats:**

Le Campus de la région de Niagara (NRC) a compté une plus grande proportion d’étudiants jumelés en médecine familiale. Pour ce qui est de l’équivalence des formations, la proportion des scores PPI se trouvant à deux écarts-types sous la moyenne était comparable sur les trois campus. L’ECOS a obtenu des différences de moins de 2 % entre les campus, mais aucune dans la dernière année de formation. Les taux de réussite à l’EACMC1 de chaque campus ne présentaient pas de différence significative, et aucune différence n’a été notée dans les taux de jumelage du CaRMS. Quant à l’efficacité de la formation, il n’y a eu aucune différence entre les taux de réussite aux trois campus obtenus à l’EACMC1 et entre les taux de jumelage du CaRMS et les taux nationaux.

**Conclusions:**

Les étudiants des trois campus ont vécu des expériences d’apprentissage équivalentes et ils se sont avérés compétents lorsque comparés aux mesures nationales. Cependant, les jumelages de résidence en médecine familiale étaient plus nombreux sur le campus de la région de Niagara. On pourrait imputer cette différence aux modèles de rôle des résidents et dirigeants ainsi qu’à l’hôpital régional et au milieu communautaire.

## Introduction

The World Health Organization (WHO) identified a commitment to social accountability as a way in which medical education, research, and service can address “priority health concerns of the community region and the nation” for the 21^st^ century.^1^ The Future of Medical Education in Canada (FMEC) MD vision articulated the call for a social accountability mission for medical schools by recommending a change in medical education to address individual and evolving community needs.^2^ With the increased demand for physician training, primary care physician training, and addressing the geographic mal-distribution of the physician workforce, many medical schools have expanded medical education outside of academic centres through distributed medical education and a Regional Medical Campus (RMC) model.^3^^-^^7^ These campuses are situated varying distances from the main campus, and offer a wide variety of the components of an undergraduate medical education.^6^^,^^7^

The two RMCs at McMaster’s Michael G. DeGroote School of Medicine, Niagara Regional Campus (NRC) and Waterloo Regional Campus (WRC) were developed in response to the Province of Ontario’s move to address physician health human resource shortages by increasing medical school enrollment during the first decade of the 21^st^ century. McMaster RMCs (NRC and WRC) provide complete pre-clerkship education with the exception of the first medical foundation curriculum which all students complete at the Hamilton Campus located at McMaster’s Academic Health Sciences Centre. The WRC site in Kitchener-Waterloo, began accepting students in 2007, and the NRC, based in St. Catharines, in 2008. These campus hubs are located 67km and 79km, respectively, from the Hamilton Campus and are surrounded by their regional campus geographic areas which support community-based clinical rotations. Core clerkship rotations are delivered primarily within the three campus regions but some rotations may occur outside the distributed campuses (see Table 1).

**Table 1:** City and regional characteristics of campuses of the M.G. DeGroote School of Medicine

	Hamilton Campus	Waterloo Regional Campus	Niagara Regional Campus
• Campus Hub City Population*	Hamilton 536,917	Kitchener-Waterloo 338,208	St. Catharines 133,113
• Region Demographics** Region Population Average Age	Hamilton 747,545 41.6	Waterloo-Wellington 757,880 39.5	Niagara 447,888 44.1
• Median Income Land Area (km^2^)	32,917 1,117	36,802 4,030	31,601 1,854
• Hospitals*** Number	6	10	7
• AHSC ≥100 beds <100 beds	Yes 6 0	No 5 5	No 3 4

* 2016 Census data for each city. https://www12.statcan.gc.ca/census-recensement/2016/dp-pd/prof/index.cfm?Lang=E

** Campus Regions defined by McMaster and corresponding demographics according to Statistics Canada Census Profile, 2016, https://www12.statcan.gc.ca/census-recensement/2016/dp-pd/prof/index.cfm?Lang=E

*** Council of Academic Hospitals of Ontario (CAHO) http://caho-hospitals.com/about-us/member-hospitals/ and hospital classification http://www.health.gov.on.ca/en/common/system/services/hosp/locations.aspx

To retain accreditation certification, medical schools in Canada must assess the efficacy of each site individually against the established standards set by the Committee on Accreditation of Canadian Medical Schools (CACMS).^8^ When establishing an RMC, schools must ensure the equivalency of the learning objectives and the methods of evaluation between all sites and provide justification for any differences.^7^ Prior to the establishment of RMCs that provided the breadth of medical training including pre-clinical education, undergraduate learners could participate in clerkship rotations in McMaster Community and Rural Education (Mac-CARE) communities.^9^ Past literature from McMaster has shown that students who elected to participate in Mac-CARE rotations performed no differently than non-Mac-CARE students, both before and after the rotation.

Interestingly, the literature suggests that medical graduates from rural training sites and RMCs are more likely to choose family medicine as a professional discipline.^10^^-^^16^ Accordingly, the primary objective of this study was to examine the way in which Canadian Residency Matching Service (CaRMS) results varied as a function of campus enrolment at McMaster’s Michael G. DeGroote School of Medicine. The fundamental idea is that medical training in contexts outside of academic tertiary care centres (including rural sites and RMCs) may promote family medicine practice because they are typically situated in communities where students receive more experience in community-based and generalist forms of medical practice; experience which is considered influential in their training^14,^^17^ and practice decisions.^18^ We wanted to test this association at our medical school.

With these notions of exposure and influence in mind, we also compared the number of family medicine practitioners that interacted with students as tutors and longitudinal facilitators at each of the campuses. Our hypothesis with these analyses was that exposure to family physicians during training may influence motivations towards family practice.

In order to ensure we were able to appropriately appraise any differences in CaRMS results as a function of campus enrolment, we also compared the learning outcomes between undergraduate learners at each of its sites. As several classes have completed their entire undergraduate education, we took the opportunity to conduct a quasi-experimental evaluation of the efficacy and equivalency of the quality of training that occurs at each of the campuses. This occurred via between-campus comparison of student performance on the Personal Progress Index (PPI) tests, Objective Structured Clinical Examination (OSCE), and the Medical Council of Canada Qualifying Examination Part 1 (MCCQE1). Establishing equivalency and efficacy of education outcomes between campuses allows us to understand the effects that RMC enrolment has on residency matching outcome above and beyond that associated with differences in academic achievement.

## Methods

The local Research Ethics Board waived the requirement for formal ethics review as the work was determined to fall under the exemption provided for quality improvement/program evaluation, as per Article 2.5 of the TCPS2 (2018).

### Sample demographics

This study compares residency match outcomes of students from McMaster’s three medical education campuses through statistical analysis of retrospective metrics. This involved review and collation of data from 972 student files (736 Hamilton Campus, 434 females, 302 males; 123 WRC, 84 females, 39 males; 113 NRC, 78 females, 35 males) associated with trainees that graduated from each campus between 2011 and 2015. The year-by-year number of graduates associated with each campus are presented in Table 2.

**Table 2:** Number of graduating students by campus by year (2011-2015)

Graduation Year	Hamilton Campus	Waterloo Regional Campus	Niagara Regional Campus
2011	145	20	14
2012	144	26	20
2013	154	26	27
2014	142	28	25
2015	151	23	27

### Family medicine residency CaRMS match proportion

The CaRMS match data that indicates to which type of program students would join in residency were categorized as either a CFPC family medicine training program or RCSPC specialty training program. The particular outcome of interest was the percentage of graduates at each McMaster Campus matching to family medicine residencies. The number of students who were matched during CaRMS first iteration into a family medicine residency, were calculated and expressed as a proportion of the matches across the five graduation years (2011-2015). These proportions were analyzed in a one-way analysis of variance (ANOVA) with campus as the only factor. We chose to examine only the CaRMs first iteration, which includes all applicants who meet the basic eligibility criteria and have no prior postgraduate training in Canada or the US. We excluded the second iteration outcomes from our analyses as these involve pairing applicants not matched in the first iteration with the remaining program availabilities. In this way, second iteration matches are often not reflective of candidate preference or intention.

### Family physician as teacher proportion

As a secondary analyses, the proportion of family physician (i.e. certified by CFPC) tutors for the Medical Foundation curriculum and the proportion of family physician longitudinal facilitators for the Professional Competencies curriculum to which student cohorts from each campus were exposed during their undergraduate training were compared using independent Yates Chi-Square analyses.

### Establishing the efficacy and equivalence of educational outcomes between campuses

In order to understand the influence of campus enrollment on the residency matching outcome, it was also necessary to determine whether there were any demonstrable differences between the educational outcomes associated to students assigned to any of the three campuses. This involved review of four sources of data: PPI scores; OSCE scores; MCCQE1 performance; and CARMs match rates.

The PPI is a 180 multiple-choice test taken by all classes in September, February, and May of each year throughout the 3-year program. A committee of clinicians and scientists developed the question bank used in the PPI. It contains questions covering all disciplines of medicine. Students are encouraged to answer only those questions to which they know the answer, and that a correction factor for guessing is applied to a computer adjusted correct score. Results are norm-referenced and students receive feedback on how the performance compares with their peers. No students would fail to progress in the program due to a poor PPI result alone. PPI test results more than two standard deviations below the class mean could lead to a focused review of the student’s knowledge base and possible remediation. The proportion of students flagged for review via PPI test scores aggregated across seven testing occasions was compared in a one-way ANOVA with Campus as the only factor. Significant effects (*p* < .05) were decomposed using Tukey’s HSD *post hoc* methodology.

The OSCE is a 10-station structured practical skills examination that is delivered once per year in McMaster’s three-year undergraduate medical program. Each station is 12 minutes in length, with nine minutes for the student to perform and three minutes for the assessor to provide feedback. The OSCE station examines history taking and physical examination skills, or sometimes, a combination of both. OSCE performances were compared in a three campus (Hamilton, Niagara, Waterloo) by five Graduation Year (2011, 2012, 2013, 2014, 2015) by three OSCE test iteration (June Year 1, November Year 2 (prior to entering clerkship), April Year 3 (following clerkship)) analysis of variance (ANOVA), with repeated measures on the last factor. Significant effects (*p* < .05) were decomposed using Tukey’s HSD *post hoc* methodology.

The MCCQE1, is a one-day, computer-based test, which assesses the competence of candidates who have obtained a medical degree for entry into supervised clinical practice and postgraduate training programs. The test assesses knowledge, clinical skills and attitudes as outlined in the Medical Council of Canada objectives.^19^ A passing grade of 390 was used until 2014 whereupon the passing grade increased to 427. The MCCQE1 pass rates across the 5-year period for each campus were compared against each other and the national pass rates over the same period via Yates Chi-Square analysis. Typically, institutions do not share raw and mean scores of qualifying examination results, therefore, only results of the analysis are reported.

CaRMS is a national, independent, not-for-profit, residency application and matching service for medical training throughout Canada.^20^ For this study, the CaRMS match rates of McMaster graduates at each campus were compared between campuses and against the national average. The proportion of graduates that successfully matched to residency programs across the 5-year period for each campus were compared against each other and the national proportions over the same period via Yates Chi-Square analysis.

## Results

### CaRMS match rate

The Yates Chi Square comparison of CaRMS match rates (Table 3) revealed no significant differences among McMaster’s three campuses, and no significant differences among the three campuses (Yates chi square (2,931) = 0.083 *p* = .95) and the national rates of success (Yates chi square (3,13632) = 0.062, *p* = .99).

**Table 3:** Mean CaRMS match rates (%) by campus and nationally (2011-2015)

CaRMS Match Rate	Hamilton Campus	Waterloo Regional Campus	Niagara Regional Campus	National Average
Percent	95.7	95.9	95.4	94.9
N	707	119	105	13,371

### Family medicine residency CaRMS match proportion

**Figure 1:**
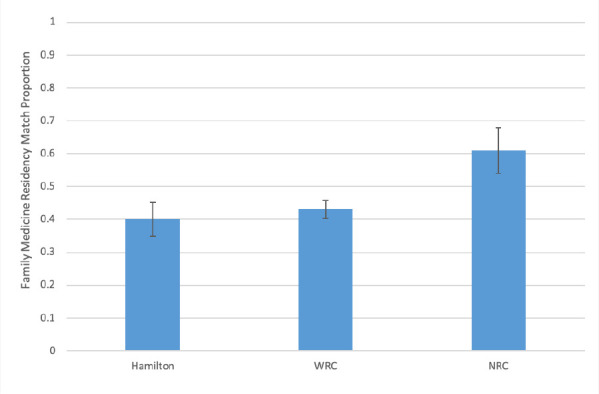
Average family medicine residency match proportion by campus (2011-2015)

There was a statistically significant difference between campuses of students who matched to family medicine, F (2, 12) = 22.15, *p* < 0.0001. *Post hoc* analysis revealed that the proportion of students matching to family medicine residencies was significantly higher for NRC (0.61 ± 0.07) compared to the Hamilton campus (0.40 ± 0.052 *, p* <0.001) and to the WRC (0.43 ± 0.027, *p* = 0.001). There were no statistically significant differences between WRC and Hamilton (*p* = 0.648). Figure 1 shows the mean match proportion to family medicine residencies and standard deviations for each of the campuses.

### Family physician as teacher proportion

The analysis revealed significant between-campus differences for the proportion of family medicine tutors who engaged with students (Yates Chi-Square (2, 841) = 373.52, *p* < .001). The proportion of tutors with CFPC certification was dramatically lower for the Hamilton campus (0.06) than for the two RMCs (Waterloo: 0.74; Niagara: 0.73).

There was no statistically significant between-campus difference for the proportion of family physician longitudinal facilitators interacted with students (grand mean = 0.63 ± 0.14; Yates Chi-Square (2, 104) = 4.35, *p* = .11).

### Efficacy and equivalence of educational outcomes between campuses

***PPI:*** The proportion of students that scored an adjusted correct percentage score more than two standard deviations below the class mean was less than 2% for each of the three campus cohorts, with a significantly smaller proportion of students performing this way at the Waterloo Regional Campus, F(2,949) = 5774.79, *p* < .001. However, we did not believe this was an educationally practical difference among campuses nor year of training (grand mean below 2SD = 1.56%/ year).

***OSCE:*** The repeated measures ANOVA of OSCE performance revealed significant main effects for campus F(2, 921) = 3.17 *p* = 0.04, *d* = 1.25, and test iteration F(2,1842) = 43.23, *p* < 0.001, *d* = 0.76. Despite these main effects, it is important to note that the analysis indicates that there were no statistically significant differences between campuses in student performance on their third and final test iteration (Table 4).

**Table 4:** Mean Year 1, Year 2, and Year 3 OSCE scores (%; SD) by campus from 2011-2015

Campus	Year 1	Year 2	Year 3
Hamilton	75.8 (5.8)	76.1 (6.4)	78.6 (5.3)
Waterloo	76.4 (5.3)	76.5 (5.5)	78.3 (5.4)
Niagara	74.4 (6.1)	75.0 (5.5)	78.0 (5.5)

***MCCQE1 analysis:*** The Yates Chi Square comparison of MCCQE1 pass rates revealed no statistically significant differences among McMaster’s three campuses, and no significant differences between the three campuses and the national rates of success (Yates chi square (3,3622) = 6.44; *p* = .09).

## Discussion

Since physician mal-distribution is most likely experienced in rural and remote regions, and with respect to primary care services, a perceived benefit to the establishment of RMCs is the production of physicians who are prepared and inclined to provide primary care services to these populations.^10^^,^^14^^,^^21^^-^^22^ This is typically understood in terms of a refined ability and particular willingness to provide primary care to those communities as a family physician.

This study investigated the relationship between enrolment in an RMC and the likelihood of a student match to CFPC-accredited residency programs. This began with an interrogation of the campus-associated factors that may promote a tendency towards a career in family medicine. The analysis revealed that NRC students were significantly more likely to match to family medicine residencies than were their counterparts in Hamilton and the WRC. Interestingly, that this effect was present for the NRC but not the WRC indicates that it is not the regional, or distributed, orientation of the campus, *per se*, that drives a greater association with family medicine residency. Presumably there is a specific feature (or features) of the Niagara campus or the students that attend the campus that produces this effect. Review of the regional characteristics (Table 1) demonstrates that there are no great differences between NRC and WRC hospital number and sizes; however, the NRC is served by one hospital system whereas WRC has seven hospital systems. There are differences in population of the campus hub city and region, and income, with the NRC region lowest for each; features more commonly found in smaller communities and rural areas. As previously mentioned, medical training in smaller communities is more likely to lead to family medicine specialty choice. We also found no differences between the two regional campuses in the amount to which learners are exposed to family physicians as educators.

Importantly, examination of the learning outcomes between the three campuses highlighted that any perceived match effect was not attributable to differences in the academic achievement of the students at each of the three campuses. By the conclusion of training, the students at each of McMaster University’s medical education (i.e. Hamilton, Niagara and Waterloo) campuses in the examined time period performed with no educationally meaningful differences on both internal (i.e.: PPI; OSCE) and Canadian (MCCQE1) examinations.

In considering additional NRC specific factors that may be at play, we recognized three that would benefit from further exploration. The first potential factor is the possible influence associated with the accreditation status (i.e., CCFP v FRCPS) of the Regional Assistant Dean at the relevant campus over the time of inquiry. In this regard, between 2011 and 2015, the administrative leader for the NRC was an academic family physician with active membership in the Society of Rural Physicians of Canada, while the leaders of the other two campuses were physicians accredited through the RCPSC. The school employs numerous policies, governance structures, and oversight to ensure consistency in academic planning. It may be that impact associated with dean-level leadership is limited. Nevertheless, we can speculate that despite the same process across campuses for the selection of learners and the delivery of curriculum the NRC may have other factors that influenced career choice in family medicine. These influences may include extra-curricular activities, career discussions with the dean and executive leadership (most of whom were family physicians during the study time period), and involvement with community organizations.

The second potential factor is the variety of the residency programs aligned with each campus. Specifically, the Hamilton campus serves as a hub for McMaster residents in over 50 different postgraduate medical training programs and the Waterloo Regional Campus hosts residents in five postgraduate programs (Family Medicine, Internal Medicine, Paediatrics, and Psychiatry); while the Niagara Regional Campus is home to McMaster residents in two postgraduate programs (Family Medicine and General Surgery). It is possible that the resident role-models to whom trainees are exposed may drive a greater tendency towards family practice because the residents are only slightly further along in their professional development trajectory. This position is consistent with literature that shows experiences with near-peer, resident-level role models is strongly associated with undergraduate medical students’ decisions about career choice.^23^As such, it is possible that an increased interaction with family medicine residents (or decreased interaction with residents in specialty programs) is a mechanism that promotes family medicine among NRC students.

The third potential factor is that the Niagara Health System of hospitals, during the period of study, came under provincial supervision, a situation bringing both concern and hope to the region’s healthcare.^24^ Perhaps a struggling hospital system in conjunction with fewer specialty resident-level role models and conversely more accessible family medicine decanal and resident-level role models, may have tipped the scales to encourage learners to choose a career in family medicine.

We also acknowledge the strong likelihood that some or all of these factors interacted with each other, in the context of any one campus, situated in any particular region, to influence learner decisions about future practice. Furthermore, we were not able to explore individual student level issues that may lead to a match in family medicine, including, their career interest prior to entering medical school, individual exposures to family medicine while in medical school through elective rotations, other life circumstances such as family pressures or age on entering medical school that may also make a career in family medicine more appealing. There may be an element of chance, or even intention, that students more interested in family medicine happened to select one RMC over another when ranking campus choice at admission. We recognize that the relationships between regional medical campuses and the choices that learners make are complex and multi-factorial. This is an area that requires deeper inquiry, an inquiry which looks beyond student performances and considers the nature of their experiences—before, within, and concurrent with their medical education. We plan to further examine these questions with an exploration of the residency match outcomes in the following five years (Class of 2016-2020).

## Conclusion

While the educational outcomes at McMaster University’s three undergraduate medical campuses were equivalent for graduates between 2011 and 2015, there was a significantly higher proportion of students from the Niagara Regional Campus that matched to CFPC-accredited residency programs during that same time period. With respect to the idea that regional training promotes the willingness to provide family medicine care to local populations, these data suggest that there is either little direct association between distributed learning and the motivation for a career in family medicine or that it is features above and beyond merely a distributed education that promote such a relationship. Given the accumulated evidence in this regard,^13^ it is our position that consideration for specific campus features deserves more attention. In acknowledging that there may be local factors (at any campus) that contribute to training that facilitate learner progress towards family medicine (or any medical discipline), we must also consider how learner assignments and faculty placements at RMCs and the distributed medical education program at McMaster University in general might be affected if we were able to identify those factors that nudged students towards a particular career in medicine.

## References

[1] Boelen C Prospects for change in medical education in the twenty-first century. Acad Med. 1995;70(7):S21-S28 10.1097/00001888-199507000-00017 7626157

[2] >The future of medical education in Canada (FMEC): a collective vision for MD education. Ottawa Association of Faculties of Medicine of Canada. 2010 Available at: https://afmc.ca/pdf/fmec/2010-FMEC-MD.pdf [Accessed Oct 6, 2019]

[3] Association of Faculties of Medicine of Canada. Mapping undergraduate distributed medical education in Canada. Ottawa, ON: AFMC 2010.

[4] Busing N, Harris K, MacLellan A, Moineau G, Oandasan I, Rourke J, et al The future of postgraduate medical education in Canada. Acad Med. 2015;90(9):1258-63. 10.1097/0000000000000815 26177532

[5] Ellaway R, Bates J Distributed medical education in Canada. CMEJ. 2018;9(1):e1-5. 10.36834/cmej.43348 PMC6104335 30140329

[6] Anderson MB, Kanter SL Medical education in the United States and Canada, 2010. Acad Med. 2010;85(9)S2-18. 10.1097/ACM.0b013e3181f16f52 20736548

[7] Cheifetz CE, McOwen KS, Gagne P, Wong JL Regional Medical Campuses: A new classification system. Acad Med. 2014;89(8)1140-3. 10.1097/ACM>0000000000000295 24826857

[8] Committee on Accreditation of Canadian Medical Schools. Available at: http://cacms-cafmc.ca/ [Accessed Oct 5, 2019]

[9] Bianchi F, Stobbe K, Eva K Comparing academic performance of medical students in distributed learning sites: the McMaster experience. Med Teach. 2008;30(1):67-71. 10.1080/01421590701754144 18278654

[10] Strasser R, Lanphear J. McCready WG, Topps MH, Hunt DD, Matte MC Canada’s new medical school: The Northern Ontario School of Medicine: Social accountability through distributed community engaged learning. Acad Med. 2009;84(10):1459-64. 10.1097/ACM.0b013e3181b6c5d7 19881443

[11] Brokaw J, Mandzuk C, Wade M, et. Al The influence of regional basic science campuses on medical students’ choice of specialty and practice location: a historical cohort study. BMC Med Educ. 2009;9(29). 10.1186/1472-6920-9-29 PMC2700105 19500392

[12] Liaw W, Cheifetz C, Luangkhot S, Sheridan M, Bazemore A, Phillips RL Match rates in family medicine among regional campus graduates, 2007-2009. JABFM. 2012 11;25(6):894-907. 10.3122/jabfm.2012.06.110336 23136330

[13] Crump WJ, Fricker RS, Ziegler C, Wiegman DL, Rowland ML Rural track training based at a small regional campus: equivalency of training, residency choice, and practice location of graduates. Acad Med. 2013;88(8):1122-8. 10.1097/ACM.0b013e31829a3df0 23807110

[14] Strasser R, Hogenbirk J, Minore B, Marsh D, Berry S, McCready W, et al Transforming health professional education through social accountability: Canada’s Northern Ontario School of Medicine. Med Teach. 2013;35(6):490-6. 10.3109/0142159X.2013.774334 23496120

[15] Lovato C, Hsu HCH, Bates J, Casiro O, Towle A, Snadden D The regional medical campus model and rural family medicine practice in British Columbia: a retrospective longitudinal cohort study. CMAJ Open 2019:E415-20. 10.9778/cmajo.20180205 PMC6588542 31227483

[16] Zink T, Centre B, Finstad D, et al Efforts to graduate more primary care physicians and physicians who will practice in rural areas: examining outcomes from the University of Minnesota-Duluth and the rural physician associate program. Acad Med. 2010;85(4):599-604. 10.1097/ACM.0b013e3181d2b537 20354374

[17] Strasser R, Neusy A Context counts: training health workers in and for rural and remote areas. Bull World Health Organ 2010:88:777-82. 10.2471/BLT.09.072462 20931063 PMC2947041

[18] Noble J, Baerlocher MO Future practice profiles of Canadian medical trainees. Clin Invest Med. 2006;29(4):208-9. 16986484

[19] Medical Council of Canada Qualifying Examination Part 1 (MCCQE1), 2019. Available at: https://mcc.ca/examinations/mccqe-part-i/ [Accessed Oct 6,2019].

[20] Candian Resident Matching Service (CaRMS), 2019. Available at: https://www.carms.ca/the-match/ [Accessed Oct 6, 2019].

[21] Schofield A, Bourgeois D Socially responsible medical education: innovation and challenges in a minority setting. Med Educ. 2010;44:263-71. 10.1111/j.1365-2923.2009.03573.x 20444057

[22] Toomey P, Lovato C, Hanlon N, Poole G, Bates J Impact of a regional distributed medical education program on an underserved community: perception of community leaders. Acad Med. 2013 6 88(6):811-8. 10.1097/ACM.0b013e318290f9c7 23619079

[23] Wright S, Wong A, & Newill C The impact of role models on medical students. Journal of General Internal Medicine, 1997, 12(1), 53-56. 10.1007/s11606-006-0007-1 9034946 PMC1497058

[24] Shearman L Trust, communication top to-do list for new Niagara Health System supervisor. Global News, 2011 Available at: https://globalnews.ca/news/149821/trust-communication-top-to-do-list-for-new-niagara-health-system-supervisor/ [Accessed Oct 20, 2019]

